# Post-treatment parenthood in Hodgkin's lymphoma survivors

**DOI:** 10.1038/sj.bjc.6603711

**Published:** 2007-04-03

**Authors:** C E Kiserud, A Fosså, H Holte, S D Fosså

**Affiliations:** 1 Unit for long term outcome, Department of Clinical Cancer Research, Rikshospitalet-Radiumhospitalet Medical Center, Oslo, Norway; 2 Cancer Clinic, Rikshospitalet-Radiumhospitalet Medical Center, 0310 Oslo, Norway; 3 University of Oslo, Oslo, Norway

**Keywords:** Hodgkin's lymphoma, chemotherapy, radiotherapy, post-treatment parenthood

## Abstract

Attempted and achieved post-treatment parenthood, with or without use of assisted reproduction techniques (ARTs), was assessed in Hodgkin's lymphoma survivors treated from 1971–1998, aged below 50 (females) or 65 (males) at diagnosis, aged 18 to 75 at survey. Four treatment groups were constructed: radiotherapy only, low -, medium - and high gonadotoxic chemotherapy (with or without radiotherapy in the three chemotherapy groups). Using Kaplan–Meier estimates, log-rank tests and Cox regression analyses, factors influencing post-treatment parenthood were investigated, with birth of the first child after treatment as the end point. Forty-five per cent (120/269) of males and 50% (91/184) of females reported attempted post-treatment parenthood. Of these, 76 (63%) males and 68 (75%) females had a child without use of ARTs. In addition 10 males and one female achieved post-treatment parenthood with use of ARTs. Treatment group was significantly associated with post-treatment parenthood, with highest probabilities after radiotherapy only and low gonadotoxic chemotherapy. In univariate analyses, age at diagnosis was a significant factor related to post-treatment parenthood in females.

Most previous studies on fertility in Hodgkin's lymphoma survivors (HLSs) have addressed post-treatment spermatogenesis and secondary amenorrhea (Viviani *et al*, 1985, 1991; Hill *et al*, 1995; Behringer *et al*, 2005). Although the number of achieved pregnancies and childbirths is sometimes reported in small series (Schilsky *et al*, 1981; Whitehead *et al*, 1983; Bonadonna *et al*, 1984; Anselmo *et al*, 1990), the selection of survivors attempting parenthood is not reported, with a few exceptions (Aisner *et al*, 1993; Hodgson *et al*, 2006).

We therefore aimed to determine rates for attempted and achieved post-treatment parenthood with or without use of assisted reproduction techniques (ARTs), in HLSs treated at the Rikshospitalet-Radiumhospitalet Medical Center (RRMC).

## PATIENTS AND METHODS

### Patients

**Figure 1:**
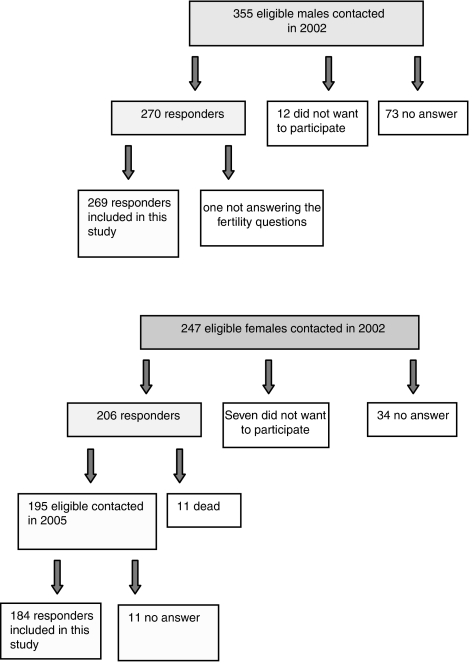
Flow charts of male and female HLSs who met the inclusion criteria for the present study.

During 1971—1998, a total of 1567 HL patients were registered in the lymphoma database of the RRMC. A questionnaire survey concerning various late effects and quality of life was performed from 2001 to 2002 among consecutive HLSs fulfilling the following criteria: treatment from 1971 to 1998, age 18–75 years at the time of survey, no relapse after 1 January 1999, no secondary cancer (except cutaneous basal cell carcinoma) and valid postal address. The present study addresses the survivors' achievement of post-treatment parenthood, as evidenced in the survey, and excluded females aged above 50 years and males above 65 years at diagnosis. Patients diagnosed with a secondary non-Hodgkin's lymphoma (NHL) were included as their treatment options were similar to those with relapsed HL. For supplementary information on fertility, the female responders from 2002 were contacted again in 2005. The present study includes male responders from the survey of 2002 and female responders from the survey of 2005 (with their reproduction as of 2002) (Figure 1).

### Treatment principles

The treatment strategies of HL at the RRMC have been described previously (Abrahamsen *et al*, 1996, 1997; Holte *et al*, 1996; Aurlien *et al*, 1998; Blystad *et al*, 2001a, 2001b) and are summarized in Table 1a and b. From 1985 to 1990, primary chemotherapy with MVPP/ChlVPP was gradually replaced by ABOD or EBVP for limited disease. At relapse, patients were treated with noncross-resistant chemotherapy, or – from 1990 –with high-dose chemotherapy with autologous stem cell support (HDT). Fractionated total body irradiation (TBI) with high-dose cyclophosphamide was used as a conditioning regimen for HDT until 1995, and was thereafter changed to chemotherapy only (BEAC/BEAM) (Blystad *et al*, 2001a, 2001b).

In stage I and II patients, mantle field and inverted Y field radiotherapy dominated up until 1997 with target doses of 41.4 Gy (1.8 Gy × 23) given alone or after chemotherapy. If possible, the standard inverted Y field was modified (to unilateral L-field or para-aortic field) in order to reduce the gonadal doses. Patients with stage III and IV received radiotherapy to areas of initial bulky tumours or residual masses after chemotherapy. Gonadal shielding was routinely used, reducing gonadal doses from 0.6 to 0.1–0.2 Gy during mantle field irradiation. Inverted Y field resulted in testicular doses of 0.6–0.9 Gy. In the 1970s, medial transposition of the ovaries was offered to young patients. In these patients, the shielded ovaries received about 3 Gy when treated with inverted Y field (Jetne, RRMC, 1972, unpublished data).

For the purpose of this study, the total treatment for each patient as registered in the lymphoma database was summarised. Chemotherapy was sub-grouped according to the expected gonadotoxicity of the regimens used: low (LowChem), medium (MedChem) and high (HighChem) gonadotoxicity (Viviani *et al*, 1985; Howell and Shalet, 2001; Behringer *et al*, 2005; American Society of Clinical Oncology *et al*, 2006; Hodgson *et al*, 2006).Two sub-groups of radiotherapy were defined: supradiaphragmatic radiotherapy (SupRad) only and infradiaphragmatic radiotherapy with or without supradiaphragmatic irradiation (InfRad). In preliminary analyses, no differences were observed between these two subgroups of radiotherapy as to attempted or achieved post-treatment parenthood. Therefore, one treatment variable (treatment) was constructed discriminating patients with radiotherapy only from those having chemotherapy with low, medium and high gonadotoxicity (with or without radiotherapy).

### Assisted reproduction techniques

The Norwegian legislation on ARTs has been rather restrictive. Since 1980, Norwegian male patients have had the option for pretreatment semen cryopreservation, with ARTs performed as intrauterine insemination (IUI) in the early 1980s and as *in vitro* fertilisation (IVF) since 1988. Intracytoplasmatic sperm injection (ICSI) has been offered since 1995. Pretreatment cryopreservation of fertilised oocytes has been offered since 1988, but has never been used by any cancer patient. Use of donated semen, but not oocytes, is allowed.

### Questionnaire, data management and ethical considerations

The questionnaire included instruments on fatigue and quality of life as reported previously (Hjermstad *et al*, 2005, 2006), in addition to questions concerning reproduction relevant for the present analysis. We assessed attempted and achieved post-treatment parenthood, number of pre- and post-treatment children, calendar years of childbirths and use of ARTs (without differentiation between IUI, IVF and ICSI and without recording the use of hormone therapy alone for inducing ovulation). Data from the questionnaire survey were merged with the lymphoma database of the RRMC.

Informed consent was obtained from all responders. The participants were informed in the invitation letter that the survey included various late effects after cancer treatment and quality of life. The Regional Committee for Medical Research Ethics, Health Region South, Norway and the Institutional Review Board at the RRMC approved the study.

### Statistics

Data were analysed using descriptive statistics (SPSS 13.0 for PC).

Factors influencing attempted post-treatment parenthood were analysed by cross-tables and *χ*^2^ tests for categorical variables, and by *t*-test for continuous variables. Binary logistic regression analyses were carried out with attempted post-treatment parenthood regarded as the dependent factor.

In patients attempting post-treatment parenthood Kaplan–Meier estimates, log-rank tests and Cox regression analyses evaluated the probability of becoming parents without use of ARTs, with the first post-treatment childbirth as the end point. The observation time for post-treatment parenthood started 8 months after start of last treatment and ended at the date of first childbirth, or 30 June 2002 (the cutoff date of the study), whichever occurred first. For Kaplan–Meier analyses, patients were categorised into three groups according to age at diagnosis: (I): ⩽20.0 years, (II): 20.1–30.0 years, (III): 30.1–40.0 years at diagnosis. In Cox regression analyses, age at diagnosis was used as a continuous variable because of lack of proportionality in the Kaplan–Meier curve. Variables significant in univariate analyses were included as covariates in multivariate analyses together with clinical parameters of major interest. *P*-values less than 0.05 (two-tailed) were considered significant, and 95% confidence intervals are given.

## RESULTS

### Patients' characteristics

Of the 1567 HL patients registered in the database from this period, the 602 eligible for the present study were contacted by mail (811 were dead at the time of the survey, 107 had a second cancer diagnosis, 24 had relapse after 1999, 19 were excluded because of age criteria and four had no valid postal address). There were 269 male and 184 female responders (a response rate of 75% (Figure 1)). No differences in observation time, initial stage, principal therapeutic group and relapse rates were observed for responders and non-responders. Responding males were significantly older than the non-responding males (29.4 *vs* 26.8 years (median)), whereas the opposite was the case in females (26.1 *vs* 32.3 years (median)).

Of all responders, 86% were <40 years at diagnosis. The median observation time from last treatment to survey was 15 years (range 3–34 years), with 62% of the patients diagnosed before 1989, and 61% treated with radiotherapy and chemotherapy. At the time of diagnosis, 46% had at least one child (127 males had 1–7 children and 83 females had 1–6 children) (Table 2).

### Attempted post-treatment parenthood

Ninety-one (50%) females and 120 (45%) males reported to have attempted post-treatment parenthood, all aged below 40 years at diagnosis. In both univariate and multivariate analyses, low age and childlessness at diagnosis were the only variables associated with post-treatment attempts of parenting. Twenty-three females reported not to have attempted post-treatment parenthood because they were convinced to be infertile after treatment; three of them were treated with radiotherapy only, 17 treated with medium or high and three with low gonadotoxic chemotherapy. The comparable number for males is not known.

### Post-treatment parenthood

**Figure 2:**
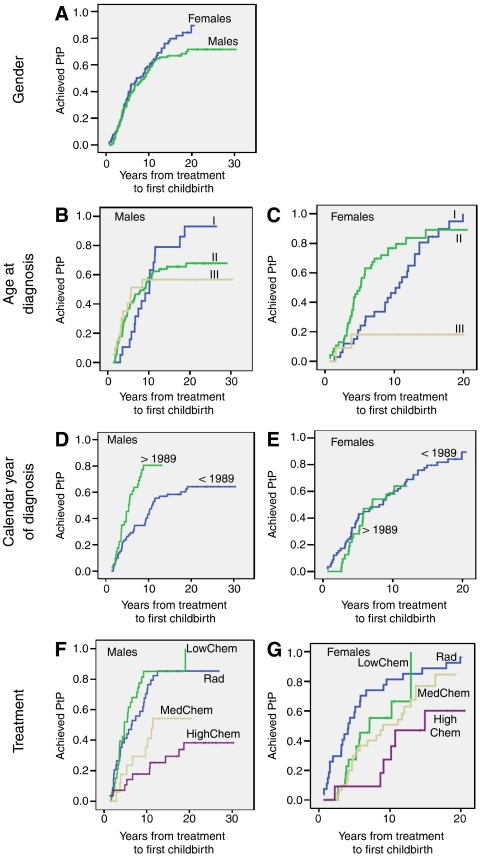
Achieved post-treatment parenthood (PtP) in male and female HLSs among those who attempted PtP, according to: (**A**) Gender. Males *n*=112, females *n*=90. (**B** and **C**) Age at diagnosis. (I) ⩽20 years (*n*=33 females, *n*=19 males), (II) 20.1–30.0 years (*n*=46 females, *n*=73 males), (III) 30.1–40.0 years (*n*=11 females, *n*=20 males). (**D** and **E**) Calendar year of diagnosis. <1989: diagnosis before 1989 (*n*=72 males, *n*=58 females), >1989: diagnosis in/after 1989 (*n*=40 males, *n*=32 females). (**F** and **G**) Treatment Rad=radiotherapy only (*n*=34 males, *n*=27 females), LowChem=low gonadotoxic chemotherapy (*n*=33 males, *n*=22 females), MedChem=medium gonadotoxic chemotherapy (*n*=17 males, *n*=30 females), HighChem=high gonadotoxic chemotherapy (*n*=28 males, *n*=11 females).

At the time of the survey, 68 females (75%) and 76 males (63%) who had attempted post-treatment parenthood had been successful without use of ARTs. The 10-year probability of post-treatment parenthood was 59% in females and 56% in males (no significant difference between genders) (Figure 2, Table 3). In patients childless at diagnosis, the 10-year probabilities of post-treatment parenthood were 58% in females and 54% in males (data not shown).

Females aged below 30 years at diagnosis were significantly more likely to achieve post-treatment parenthood compared to older females. After 10 years, the group aged 20.1–30.0 at diagnosis had the highest probability of post-treatment parenthood: 77%, compared to 50% in the youngest group and 18% in the oldest group. Fifteen years after diagnosis, the comparable figures were 89% in the group aged 20.1–30.0 at diagnosis and 85% in those aged ⩽20 years at diagnosis. Only two of 11 females older than 30 years at diagnosis achieved motherhood, one after mantle field irradiation only and one after four EBVP followed by mantle field irradiation. In males, there was no significant difference between the age groups.

Males, but not females, diagnosed after 1988 had significantly improved probability of achieving post-treatment parenthood compared to those diagnosed before 1989, with 10-year probabilities of 81 *vs* 43%. In males, but not in females, stage I/II was followed by significantly higher probability of post-treatment parenthood, compared to stage III/IV.

In males, there was a significantly higher probability of post-treatment fatherhood after radiotherapy only and after chemotherapy of low gonadotoxicity compared to chemotherapy with medium or high gonadotoxicity. The 10-year probabilities of achieving post-treatment parenthood in the various groups were 71 (Rad), 85 (LowChem), 35 (MedChem) and 18% (HighChem).

In females, a significantly higher probability of achieving post-treatment motherhood was observed after radiotherapy only compared to chemotherapy with either medium or high gonadotoxicity. In addition, a comparable difference was seen between those treated with low and high gonadotoxic chemotherapy. The 10-year probabilities were 82% (Rad), 55% (LowChem), 51% (MedChem) and 27% (HighChem).

Treatment with highly gonadotoxic chemotherapy did not exclude the possibility of having a child without the use of ARTs. Of 11 females attempting motherhood after highly gonadotoxic chemotherapy, six were successful; all six were aged <25 years at diagnosis. The five patients who did not achieve post-treatment parenthood were 30–36 years at diagnosis (Table 4). Ten of 28 males attempting fatherhood after highly gonadotoxic chemotherapy became fathers. Two of these achieved children spontaneously 4 years after HDT with BEAM as conditioning regimen, one of them after having a first post-treatment child with the use of pretreatment cryopreserved semen.

Of all irradiated patients, 76% had received supradiaphragmatic radiotherapy only. Of 16 males and eight females who had attempted parenthood after pelvic irradiation, 11 males (four: inverted Y field; four: mantle field/inverted Y field; three: mantle field/L-field) and five females (four: L-field; one: inverted Y field preceded by oophoropexy) were successful.

In the Cox regression multivariate analysis, post-treatment parenthood was significantly associated with treatment group in both males and females. In addition, the period of diagnosis was a significant factor in males.

### Use of ARTs, gamete donation and adoptions

Thirteen men used ARTs with pretreatment cryopreserved semen, with 10 becoming fathers. Two women used IVF/ICSI, with one of them giving birth to one child.

Thus, at the end of the observation time, 69 (76%) of the females and 85 (71%) of the males who had attempted post-treatment parenthood had become biological parents after treatment, females having a mean of 1.83 children (range 1–4) and males 1.73 children (range 1–5) post-treatment.

In addition, three males achieved fatherhood with use of donor sperm cells, whereas one female had used oocyte donation (in another Nordic country) without success. Four females and 11 males had adopted a total of 24 children (range 1–3).

## DISCUSSION

Our study is the first to investigate long-term post-treatment parenthood in a large series of consecutive HLSs. About 47% of the HLSs in this study had attempted post-treatment parenthood. Young age and childlessness at time of diagnosis were significantly associated with attempts to conceive children after treatment. Of those who had attempted post-treatment parenthood, 68% became parents spontaneously. In addition, 10 males became fathers with use of pretreatment cryopreserved semen and one female conceived with use of ARTs. In multivariate analyses, type of treatment was significantly associated with achievement of post-treatment parenthood in both genders with highest probabilities after radiotherapy only and low gonadotoxic chemotherapy. In addition, an independent and significantly higher success rate was observed in males diagnosed after compared to before 1989.

Several studies have dealt with post-treatment gonadal function in HLSs, most of them with the end point of secondary amenorrhoea, spermatogenesis mirrored by sperm count analyses and the level of serum FSH (Viviani *et al*, 1985; Viviani *et al*, 1991; Hill *et al*, 1995; Behringer *et al*, 2005). For males, these analyses are a relatively appropriate parameter of fertility, whereas regular post-treatment menstruation does not equal ovulation and the possibility of pregnancy. Some authors have reported the number of childbirths achieved in HLSs, but often in small series (Schilsky *et al*, 1981; Whitehead *et al*, 1983; Bonadonna *et al*, 1984; Anselmo *et al*, 1990), and rarely with those attempting post-treatment parenthood as a denominator (Aisner *et al*, 1993; Hodgson *et al*, 2006).

Compared to these reports, our study has several methodological strengths besides its large size: our end point is the first post-treatment childbirth. Secondly, we relate our end point to the number of patients reported having attempted parenthood. For male HLSs, the proportion of 45% who reported attempted parenthood is comparable to that observed in two studies of testicular cancer survivors (TCSs), where 31 and 39%, respectively, had tried to father a child following treatment (Rieker *et al*, 1990; Brydoy *et al*, 2005).

However, our number of HLSs who have attempted post-treatment parenthood is probably underestimated for several reasons. Firstly, only surviving patients reported their attempts at parenthood. In studies on future plans for paternity in TCSs, 67% could not exclude post-treatment fatherhood at the time of diagnosis and 56% of cancer survivors responded that they would like to have a child in the future, both numbers being higher than in our study (Aass and Fossa, 1988; Schover *et al*, 1999). The difference may in part be owing to psychological mechanisms in those patients who did not succeed in having children, and expressing that they did not want children may thus be a part of a coping strategy. Furthermore, we have not systematically assessed the reasons why individual patients did not attempt to have children nor how hard the others tried to become a parent. This note of caution is supported by answers from 23 females who stated that they had not attempted post-treatment motherhood because they were convinced of being infertile after cancer treatment. Similar attitudes might be considered in females with premature ovarian failure and in males after intensive treatment.

Reported rates of parenthood after treatment for cancer will depend on selection criteria. In the present study, of those who had attempted post-treatment parenthood, 68% conceived children spontaneously. As the proportion of patients who reported to have attempted parenthood may be underestimated, our reproduction rates may be too favourable. A study of 43 premenopausal female and 51 male HLSs attempting conception after treatment reported a significantly higher frequency of achieved pregnancies in females than in males, 81 and 49% respectively. The higher pregnancy rate in females may have been overestimated because HLSs with early menopause may have been excluded from the study (Aisner *et al*, 1993). The importance of selection bias is emphasised by the figures of a registry-based study from our group, showing a 20-year probability of first post-treatment childbirth of 8% in female and 28% in male lymphoma patients aged 15–45 years at diagnosis, if all patients treated at the RRMC are included in the denominator (Fossa and Magelssen, 2004). The relatively high grade of success in achievement of post-treatment parenthood in our study may also be explained by the high proportion of patients (62%) with initial stage I/II and only 12% treated for relapse.

In both males and females, achievement of post-treatment parenthood was associated with treatment, with the type and intensity of chemotherapy being most important. This corresponds to earlier studies on male fertility after treatment for HL, with higher rates of preserved fertility after treatment with ABVD-like regimens compared to MOPP-like regimens (Viviani *et al*, 1985; Anselmo *et al*, 1990; Viviani *et al*, 1991; Hill *et al*, 1995). Two male patients spontaneously fathered children after HDT. A recent report found post-treatment conceptions in 7% of males after HDT for HL and NHL, but neither reported the use of ARTs nor attempts of post-treatment parenthood (Carter *et al*, 2006). Only a small proportion of the HLSs in this study used their pretreatment cryopreserved semen, which has also been shown for other cancer survivors (Ragni *et al*, 2003). However, 10 of 13 males successfully achieved fatherhood with their cryopreserved semen, which is more promising than in TCSs (Magelssen *et al*, 2005).

Earlier reports have shown that female post-treatment fertility depends on the degree of gonadotoxicity of the treatment given and age at diagnosis (Schilsky *et al*, 1981; Whitehead *et al*, 1983; Bonadonna *et al*, 1984; Behringer *et al*, 2005). In a recent publication, the 12-month pregnancy rate was 70% in 36 female HLSs attempting pregnancy after treatment with ABVD, with no significant difference compared to the control group (*n*=29) (Hodgson *et al*, 2006). In our study, age at diagnosis in females was a significant factor associated with post-treatment parenthood in univariate analysis. Age determines the remaining oocyte reserve (Faddy *et al*, 1992), so the probability of sufficient oocyte reserve decreases with increasing age.

No differences were found in post-treatment parenthood after supradiaphragmatic radiotherapy only compared to infradiaphragmatic radiotherapy with or without supradiaphragmatic irradiation. However, almost 80% of the irradiated patients had received supradiaphragmatic radiotherapy only, and the group of HLSs who had attempted post-treatment parenthood after infradiaphragmatic radiotherapy was too small for valid statistical comparison. As shown previously, successful pregnancies are possible even after pelvic irradiation if oophoropexy has been performed before inverted Y field irradiation (Le *et al*, 1976) or the radiation field did not include both ovaries.

The improved probability of post-treatment parenthood in males diagnosed after 1989 compared to before 1989 is as expected, owing to changes in the treatment of HL in late 1980s with introduction of fertility-saving treatment as less gonadotoxic chemotherapy and less extensive radiation fields. Changes in attitudes towards life after treatment for HL and a more optimistic view among health professionals with regard to having children after cancer treatment in the latter period may also have contributed to the increased paternity rate.

Our study has some limitations: we do not know at what time after treatment the HLSs started attempting parenthood, how seriously they attempted this goal or the fertility status of our patient's partners. Finally, we assume that each male HLS who reported spontaneous post-treatment fatherhood is the biological father of the child. Exclusion of patients with relapse after 1999 or a second cancer from the survey and the lack of relevant information in deceased patients may have led to loss of information about patients trying to have children in the years between initial treatment and relapse, second cancer or death.

Information on fertility issues is important in clinical oncological practice, and fertility-saving tasks should be discussed with patients at risk of post-treatment infertility. Females should be informed that both the treatment and their age at treatment influence their fertility potential. Females aged above 30 years at diagnosis are at high risk of becoming infertile. They constitute a subgroup for whom cryopreservation of ovarian tissue should be considered. As males have the opportunity for pretreatment cryopreservation of semen and as spermatogenesis recovers in most of them, their potential infertility after treatment is easier to deal with.

## Figures and Tables

**Table 1:** Treatment of Hodgkin's lymphoma at the RRMC 1970-1998

	**Chemotherapy**	**Radiotherapy (RT)**	**Dose and fractions of radiotherapy**
*(a) Treatment principles*			
*Stage I & II*			
1970–1980		RT only Extended fields MF/Inv Y	2 Gy × 20
1980–1988	With risk factors: four MVPP/ChlVPP before RT	Extended fields MF/Inv Y	2 Gy × 20, from 1982 1.8 Gy × 23
1988–1997	With risk factors: 2–4 EBVP before RT	Extended fields MF/Inv Y	1.8 Gy × 23

*Stage III and IV*			
1970–1985	Eight MVPP/ChlVPP	To bulky tumor or residual mass TNI to some stage III patients until 1980	2 Gy × 20
1985–1991	Eight ChlVPP or 8 ABOD/ChlVPP (alternating)	To bulky tumor or residual mass	2 Gy × 20 or 1.8 Gy × 23
1992–1998	8 ABVD	To bulky tumor or residual mass	2 Gy × 20 or 1,8 Gy × 23


**Low**	**Medium**	**High**	
*(b) Chemotherapy and expected gonadal toxicity*			
ABO(V)D	ChlVPP	ChlVPP	
EBVP	MVPP	MVPP	
	Medium if ⩽4 courses	High if >4 courses	
	CHOP	High-dose chemotherapy with autologous stem cell support BEAC/BEAM	
	MIME		

MF=mantle field; RRMC=Rikshospitalet-Radiumhospitalet Medical Center; Inv Y=inverted Y field; TNI=total nodal irradiation.

For details on chemotherapy: Aurlien *et al* (1998); Diehl (2001); Mills *et al* (1995).

**Table 2:** Patients characteristics

	**Male**			**Female**		
	**All *N*=269% are within this group**	**Attempted PtP *n*=120% are within this group**	**Not attempted PtP *n*=149% are within this group**	**All *N*=184% are within this group**	**Attempted PtP *N*=91% are within this group**	**Not attempted PtP *N*=93% are within this group**
Age at diagnosis^a^(years)^b^	29.4 (14.6–62.2)	24.4 (16.0–38.3)	34.5 (14.6–62.2)	26.1 (9.1–49.0)	23.0 (9.1–37.1)	31.1 (13.4–49.0)
Age at survey^a^ (years)	47.0 (22.0–73.9)	42.0 (25.0–61.0)	51.9 (22.0–73.9)	43.8(20.9–72.9)	39.3 (20.9–62.9)	49.1 (22.9–72.9)
Observation time^a^ (years)^c^	14.6 (2.8–30.6)	16.2 (2.8–30.0)	13.3 (3.5–30.6)	15.6 (2.9–34.1)	15.6 (3.1–34.1)	15.9 (2.9–29.3)

Parenthood at diagnosis^d^	127 (47%)	30 (26%)	97 (65%)	83 (45%)	23 (25%)	60 (65%)
Childless at diagnosis	132 (49%)	86 (72%)	46 (31%)	98 (53%)	67 (74%)	31 (33%)

Stage I and II	170 (63%)	70 (58%)	100 (67%)	112 (61%)	57 (63%)	55 (59%)
Stage III and IV	99 (37%)	50 (42%)	49 (33%)	72 (39%)	34 (37%)	38 (41%)
Relapse	36 (13%)	10 (8%)	26 (17%)	18 (10%)	7 (8%)	11 (12%)

*Treatment*						
Rad	73 (27%)	34 (28%)	39 (26%)	48 (26%)	27 (30%)	21 (23%)
LowChem	72 (27%)	33 (28%)	39 (26%)	37 (20%)	22 (24%)	15 (16%)
MedChem	63 (23%)	23 (19%)	40 (27%)	63 (34%)	31 (34%)	32 (34%)
HighChem	61 (23%)	30 (25%)	31 (21%)	36 (20%)	11 (12%)	25 (27%)
HDT	8 (3%)	5 (4%)	3 (2%)	8 (4%)	1 (1%)	7 (7%)

*Radiotherapy*						
(±chemotherapy)	*N*=238% are within this group	*N*=105% are within this group	*N*=133% are within this group	*N*=160% are within this group	*N*=83% are within this group	*N*=77% are within this group
SupRad	177 (74%)	82 (78%)	95 (71%)	125 (79%)	66 (80%)	59 (77%)
InfRad	61 (26%)	23 (22%)	38 (29%)	35 (21%)	17 (21%)	18(23%)
Pelvic radiotherapy^e^ (±supradiaphragmatic radiotherapy)	48 (20%)	16 (15%)	32 (24%)	22 (13%)	8 (10%)	14 (18%)

HDT=high-dose chemotherapy with autologous stem cell support; HighChem=high gonadotoxic chemotherapy (±radiotherapy); InfRad=infradiaphragmatic radiotherapy±supradiaphragmatic radiotherapy; LowChem=low gonadotoxic chemotherapy (±radiotherapy); MedChem=medium gonadotoxic chemotherapy (±radiotherapy); PtP=post-treatment parenthood; Rad=radiotherapy only; SupRad, supradiaphragmatic radiotherapy only.

a Median (range).

b Significant factor with regard to attempted PtP. In both males and females, *P*<0.001 in univariate analyses (*t*-test), *P*<0.001 in multivariate analyses.

c Observation time: time from diagnosis added 8 months, to time of the survey.

d Significant factor with regard to attempted PtP. In both males and females, *P*<0.001 in univariate analyses (*χ*^2^ test), *P*=0.027 in multivariate analyses.

e Pelvic radiotherapy: inverted Y-, L-, Sacrum(skeletal)- fields, total body irradiation (TBI).

**Table 3:** 10 years probability of achieving PtP among those who attempted, excluding those achieving PtP with the use of assisted reproduction techniques

	**Males *N*=112**				**Females *N*=90**			
**Variable**	**10 years prob. of PtP (%)**	**95% CI**	***P*-value (log-rank test)**	***P*-value (Cox analysis)**	**10 years prob. of PtP (%)**	**95% CI**	***P*-value (log-rank test)**	***P*-value (Cox regression analysis)**
All	56	47–65			59	48–69		
*Age at diagnosis (years)*								
(I) ⩽20.0	53	30–76			50	33–67	0.035 *vs* II 0.001 *vs* III	
(II) 20.1–30.0	58	47–69	NS	Ns^a^	77	65–89	0.001 *vs* III	Ns^a^
(III) 30.1–40.0	57	35–79			18	0–41		

*Period of diagnosis*								
< 1989	43	32–55	<0.001	0.024	59	46–71	Ns	Ns
⩾1989	81	68–93			58	40–76		

*Stage*								
Stage I and II	65	54–76	0.02	Ns	64	51–77	Ns	Ns
Stage III and IV	42	27–57			50	33–67		

Treatment				0.001				0.007
Rad	71	55–86	0.012 *vs* MedChem <0.01 *vs* HighChem		82	67–96	0.02 *vs* MedChem 0.003 *vs* HighChem	
LowChem	85	72–98	Ns *vs* Rad 0.02 *vs* MedChem <0.001 *vs* HighChem		55	34–77	Ns *vs* Rad Ns *vs* MedChem 0.048 *vs* HighChem	
MedChem	35	13–58			51	33–69		
HighChem	18	4–32			27	1–54		

HighChem=high gonadotoxic chemotherapy; lowChem=low gonadotoxic chemotherapy; MedChem=medium gonadotoxic chemotherapy; NS=not significant; PtP=post-treatment parenthood; Rad=Radiotherapy only

a Age at diagnosis as continuous variable in Cox regression analysis.

Treatment: variable discriminating patients treated with radiotherapy only from those having low, medium or high gonadotoxic chemotherapy.

**Table 4:** Children conceived by female HLSs treated with high gonadotoxic chemotherapy, for those who had attempted post-treatment parenthood (*N*=11)

**Patient no.**	**Year of diagnosis**	**Age at diagnosis (years)**	**Chemotherapy**	**Radiotherapy (RT)**	**Achieved PtP**	**Age at birth of first child post-treatment (years)**	**No of children post-treatment**
1	1976	9	Six MVPP	SupRad^a^+Paraaortic field	Yes	24	Three
2	1979	18	Six MVPP	SupRad	Yes	29	Two
3	1988	17	Six ChlVPP 2 ABOD	SupRad	Yes	27	Two
4	1976	16	Six MVPP	SupRad+Para-aortic field	Yes	33^b^	Two
5	1981	23	Eight ChlVPP	No RT	Yes	32	Two (twins)
6	1988	19	Eight ChlVPP	SupRad	Yes	28	One
7	1988	36	Eight ChlVPP	No RT	No		
8	1990	35	HDT^c^	SupRad	No		
9	1978	31	Six MVPP	SupRad	No		
10	1971	32	Eight MVPP8 CCNU po	Th10-12 3200 rtg/10 days	No		
11	1978	30	Six MVPP	SupRad+Paraaortic field	No		

a SupRad: supradiaphragmatic radiotherapy.

b First child after RT only at 18 years, relaps at 19 years, second child at age 33 years.

c HDT: high-dose chemotherapy with autologous stem cell support.
